# Supplementary material for: Th1/Th2 Immune Imbalance in the Spleen of Mice Induced by Hypobaric Hypoxia Stimulation and Therapeutic Intervention of Astragaloside IV
Source: Int J Mol Sci. 2025 Mar 13;26(6):2584. doi: 10.3390/ijms26062584 (PMC11942621; doi:10.3390/ijms26062584)
Supplement: Supplementary file 1 [file ijms-26-02584-s001.zip › Supplementary file/Supplementary file.pdf]

Supplementary Table 1. Sequences of primers used in qRT−PCR.

**Gene**	**5′−3′**	**3′−5′**
Runx1	GCAGGCAACGATGAAAACTACT	GCAACTTGTGGCGGATTTGTA
Runx3	CAGGTTCAACGACCTTCGATT	GTGGTAGGTAGCCACTTGGG
Il12rb2	AGAGAATGCTCATTGGCACTTC	AACTGGGATAATGTGAACAGCC
H2-Ab1	AGCCCCATCACTGTGGAGT	GATGCCGCTCAACATCTTGC
H2-Ob	*AGGCGGACTGTTACTTCACC*	*ATCCAGGCGTTTGTTCCACTG*
Tbx21	*AACCGCTTATATGTCCACCCA*	*CTTGTTGTTGGTGAGCTTTAGC*
Gata3	*CTCGGCCATTCGTACATGGAA*	*GGATACCTCTGCACCGTAGC*
Cd247	*GGGAGGCAAACAGAGGAGG*	*CTGGGAGGCTAAGAGGCTTC*
H2-Oa	*TCTACCAATCTTACGACGCTTCT*	*CACACGACCTCCTCGTTCT*
Il12a	*CTGTGCCTTGGTAGCATCTATG*	*GCAGAGTCTCGCCATTATGATTC*
Il4ra	*TCTGCATCCCGTTGTTTTGC*	*GCACCTGTGCATCCTGAATG*
H2-Aa	*TCAGTCGCAGACGGTGTTTAT*	*GGGGGCTGGAATCTCAGGT*

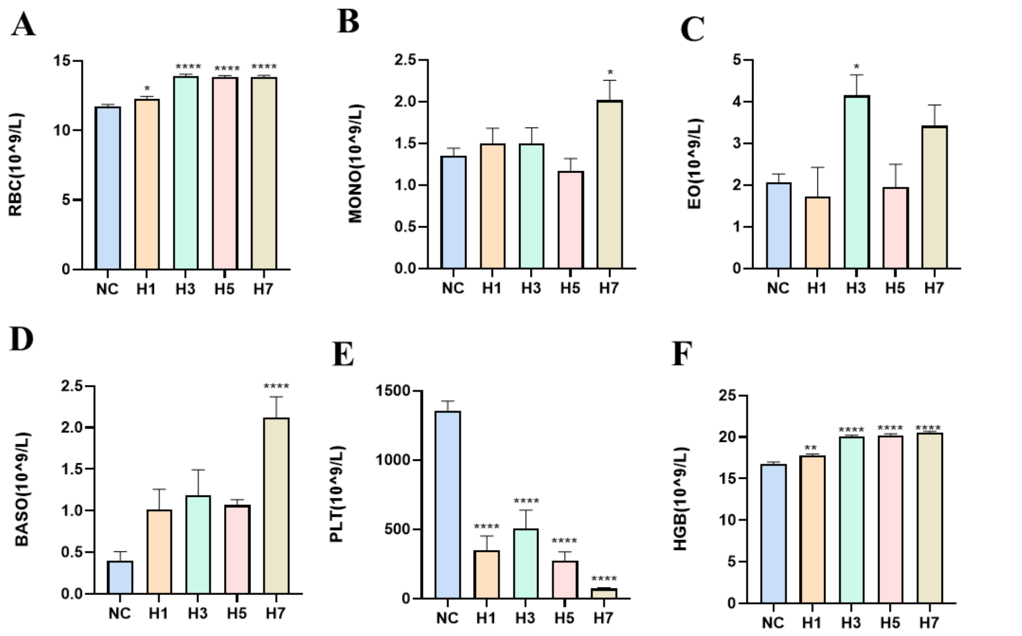

Supplementary Fig.1. Effects of different hypobaric hypoxia durations on biochemical indexes. A-F. Changes of main indexes of blood routine tests (n=4−6).

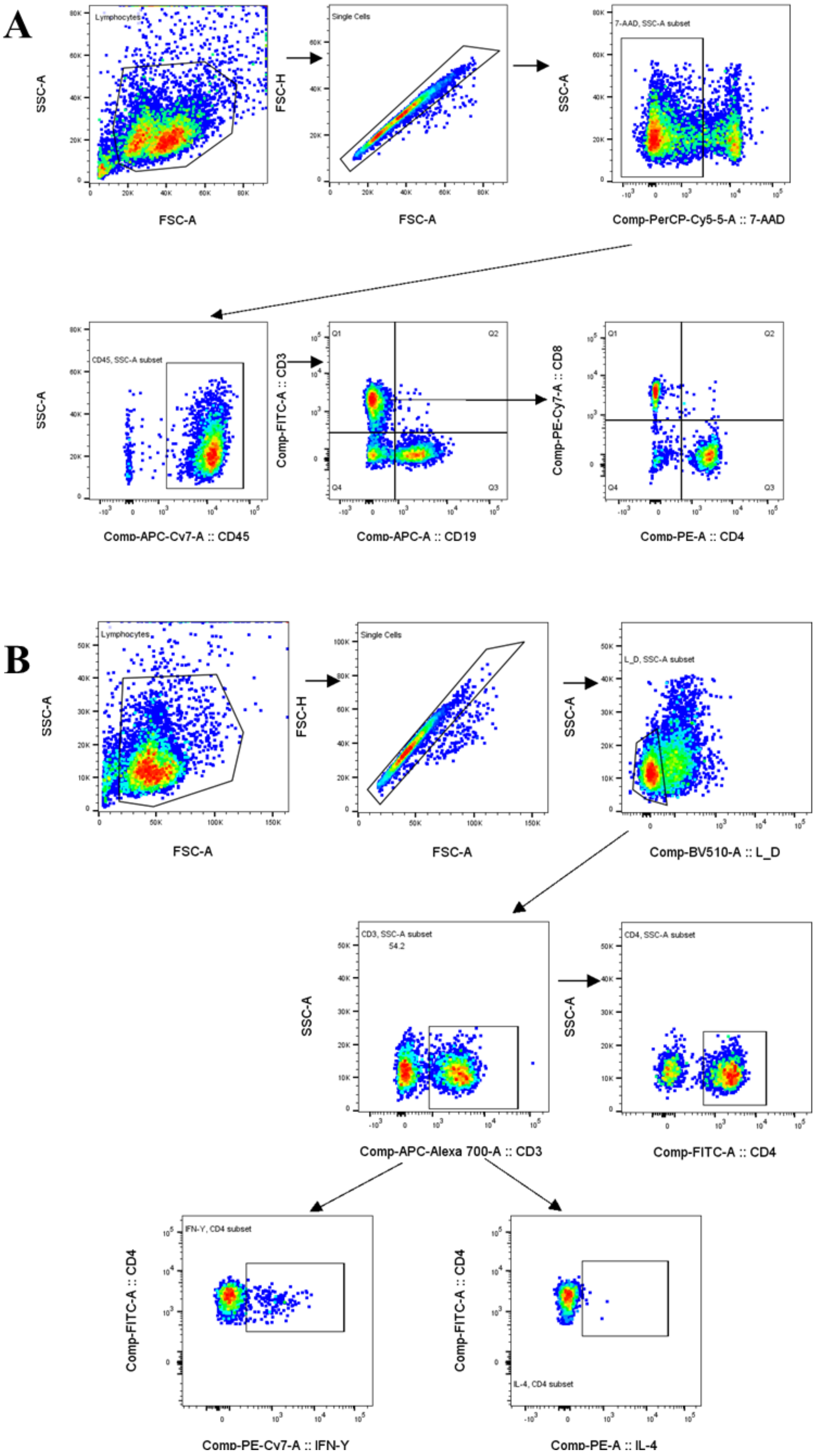

Supplementary Fig. 2. Flow gating strategy. A. Splenic lymphocytes flow gating strategy; B. Splenic Th1/Th2 lymphocytes flow gating strategy.

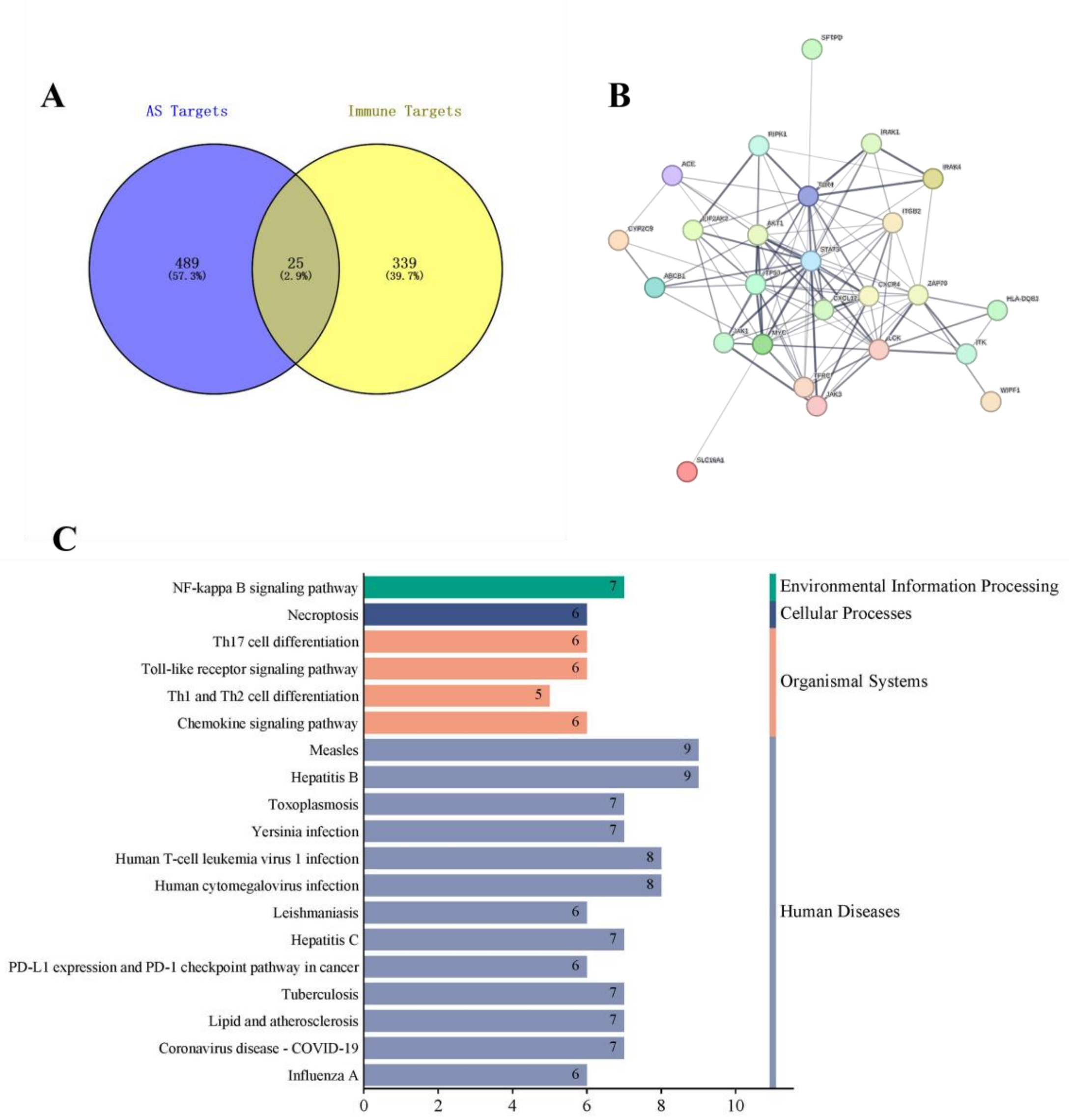

Supplementary Fig. 3. The targets of AS−IV and highland immunity were screened and the KEGG pathway gene enrichment analysis was performed. A. The common targets of saponins of AS−IV and immune injury were identified. B. The common targets were established in PPI network. C. KEGG analysis of the enriched signaling pathway of the common targets.

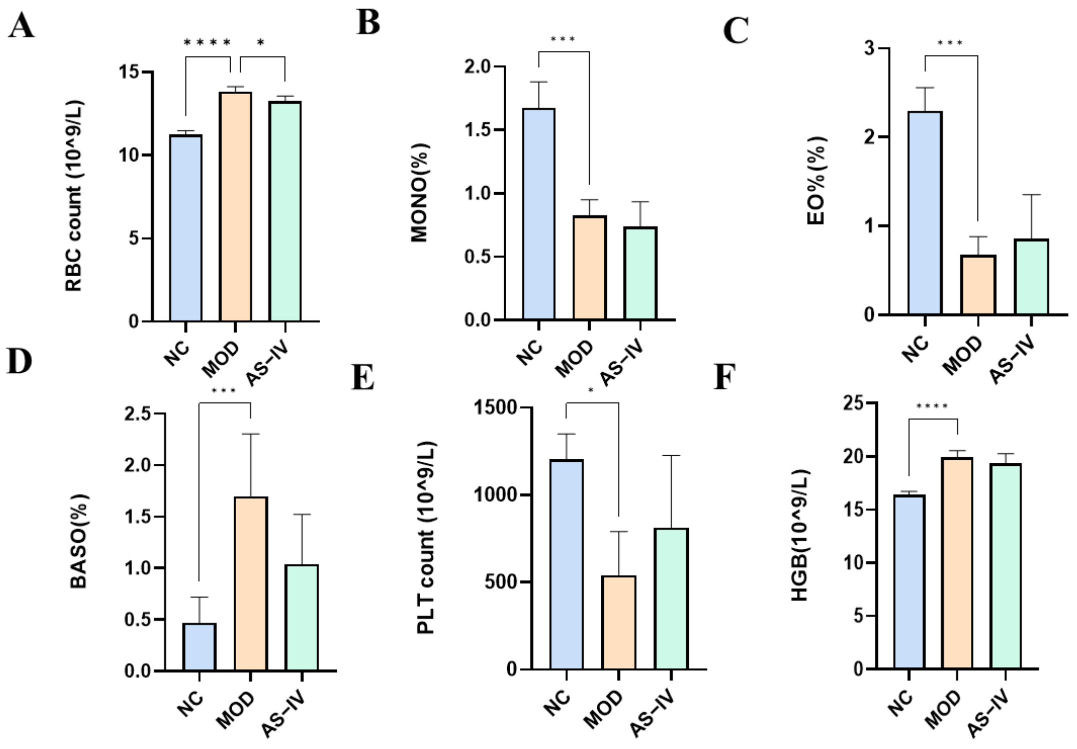

Supplementary Fig. 4. Effects of AS−IV blood routine in mice with high altitude immune injury. A−F. Changes of main indexes of blood routine tests (n=4−6).

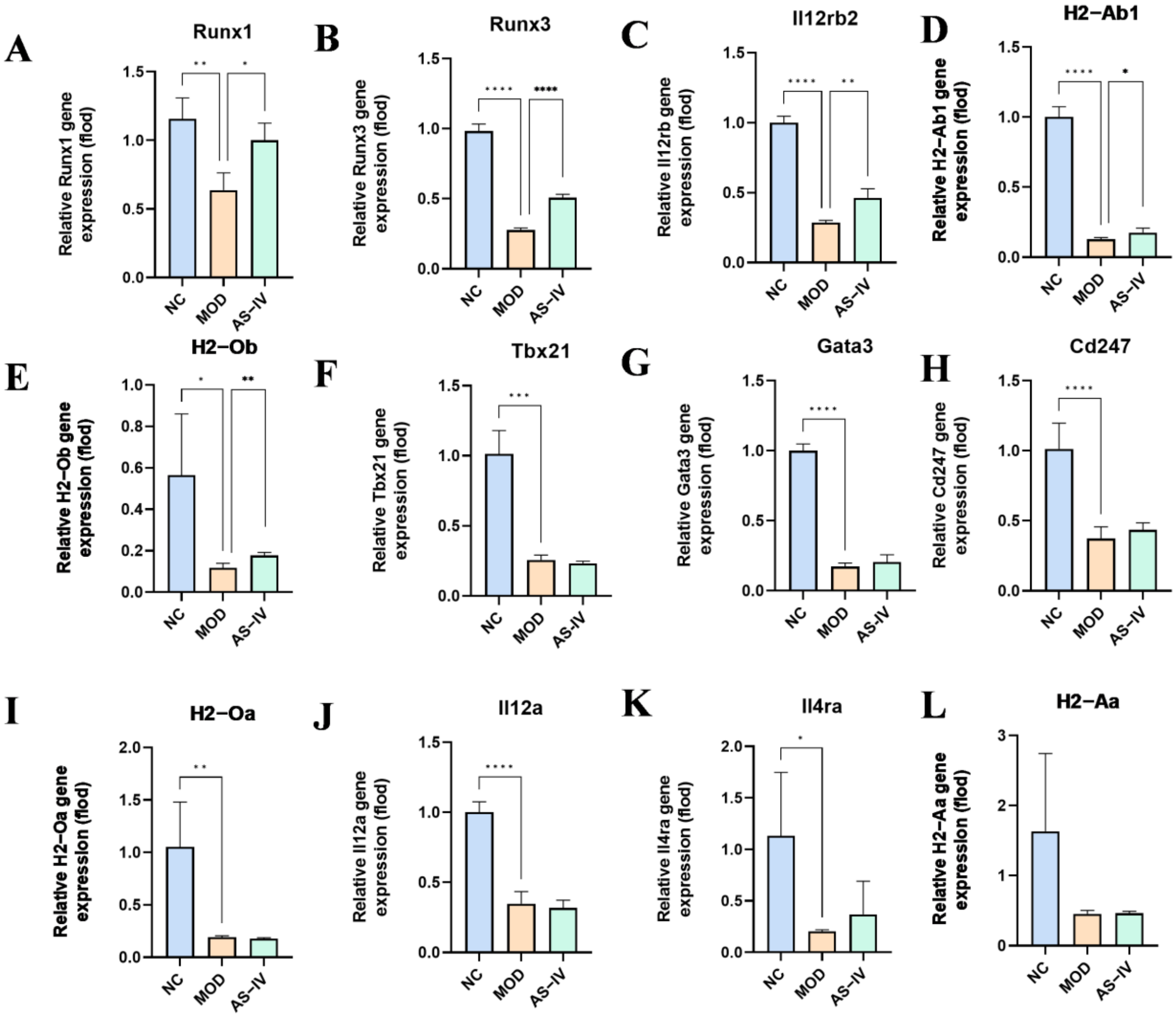

Supplementary Fig. 5. Effects of spleen mRNA in mice with high altitude immune injury. A−L. AS−IV influenced the mRNA of master transcription factors and cytokine concentrations for Th1 /Th2 (n=3).

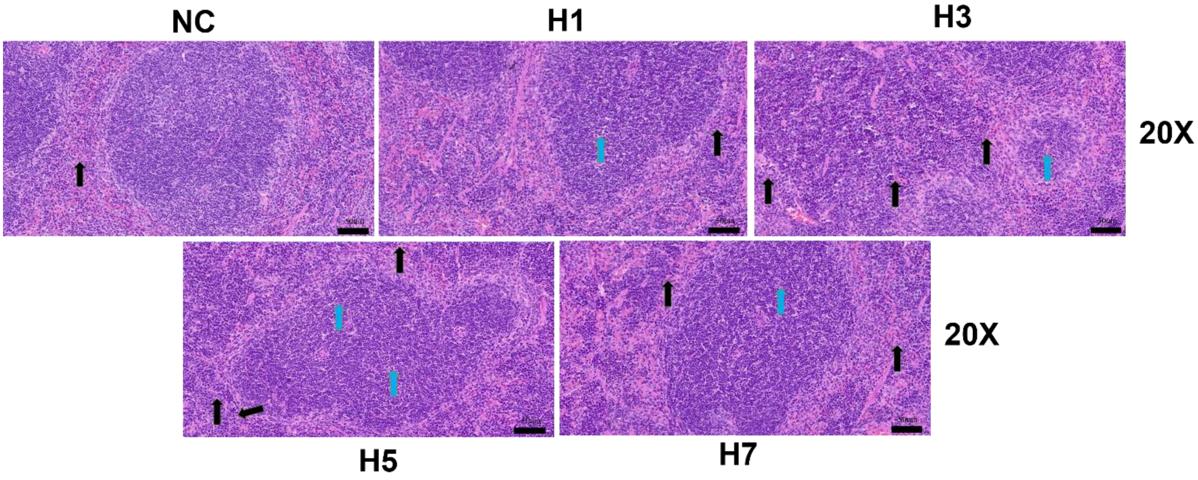

Supplementary Fig. 6. The high-resolution image of Figure 2D.

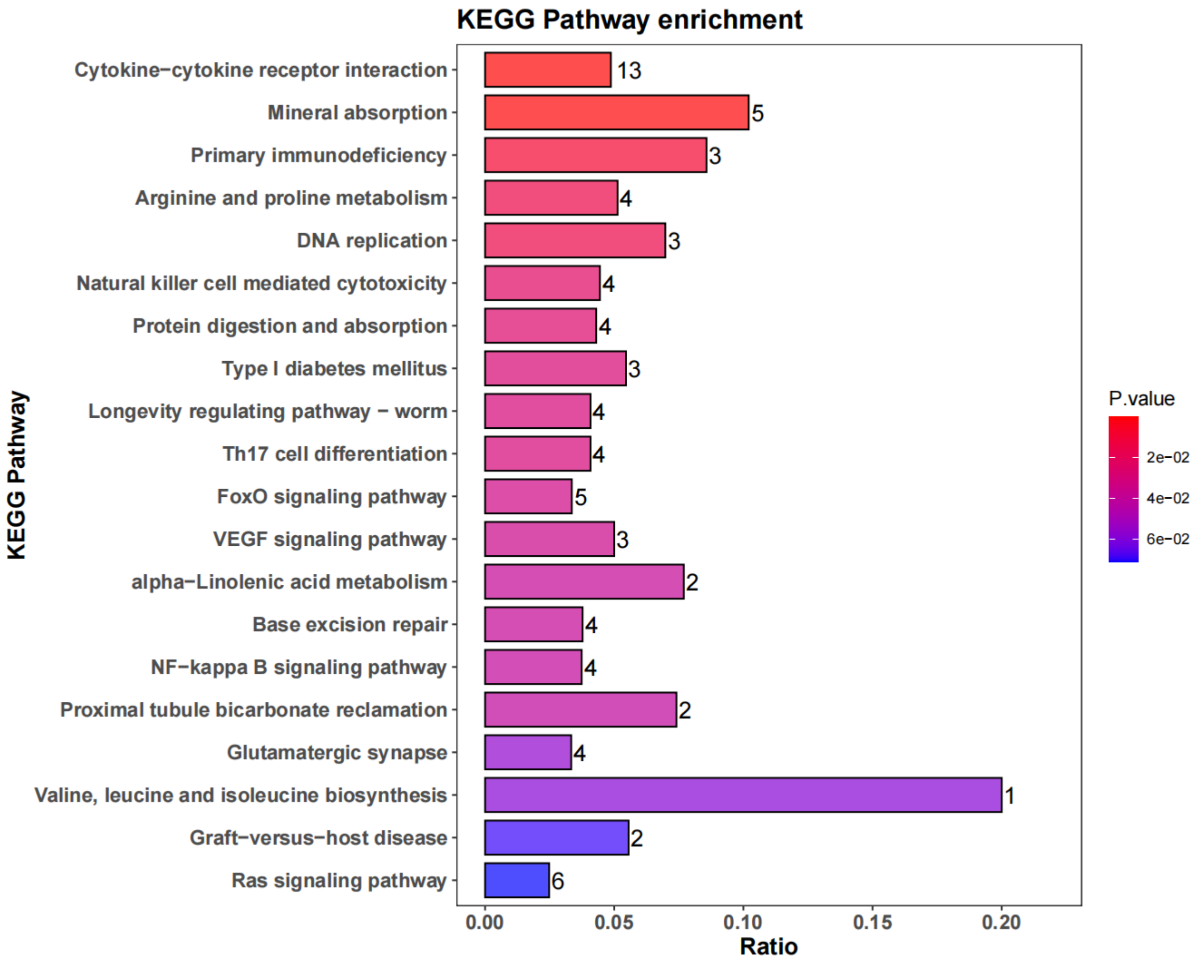

Supplementary Fig. 7. The high-resolution image of Figure 4G.

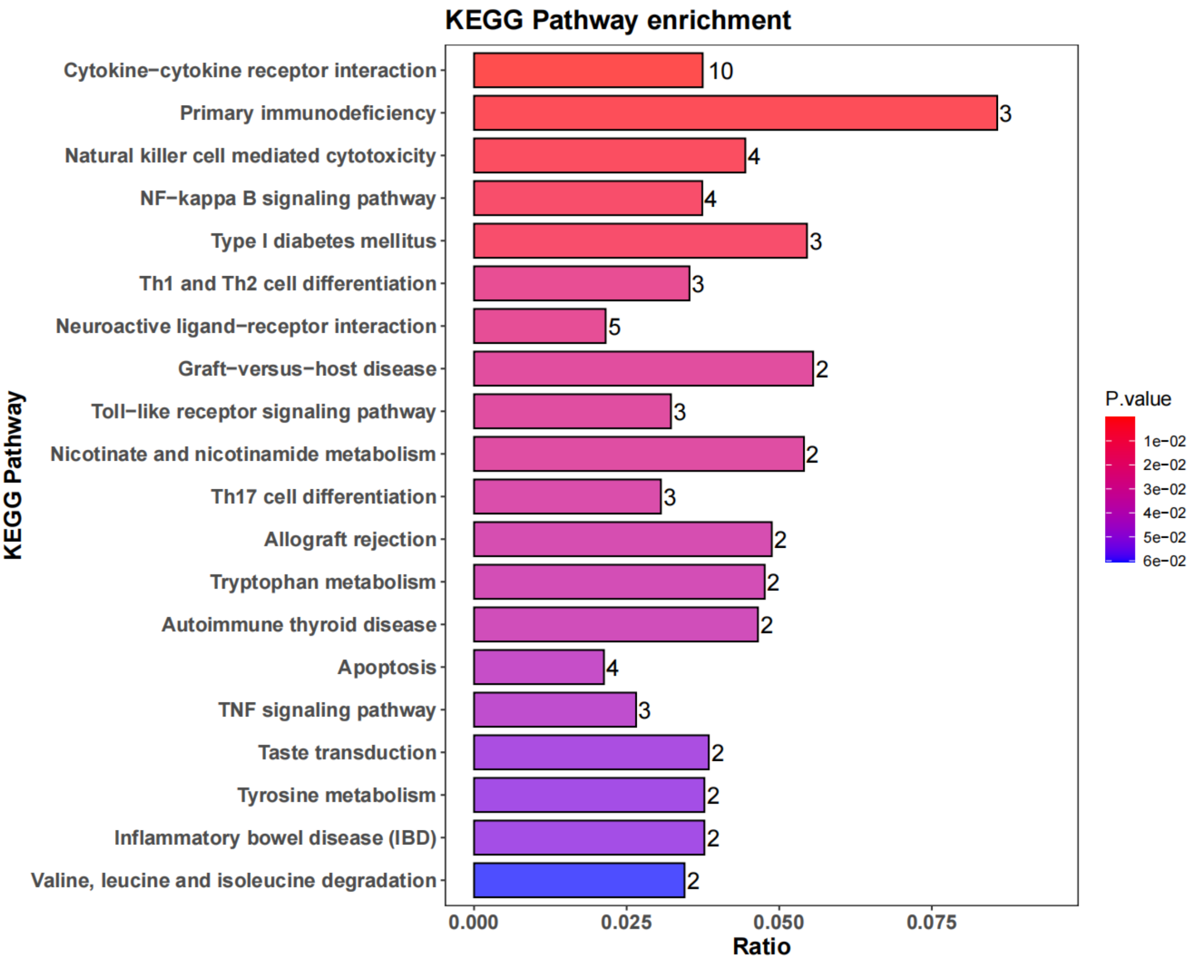

Supplementary Fig. 8. The high-resolution image of Figure 4H.

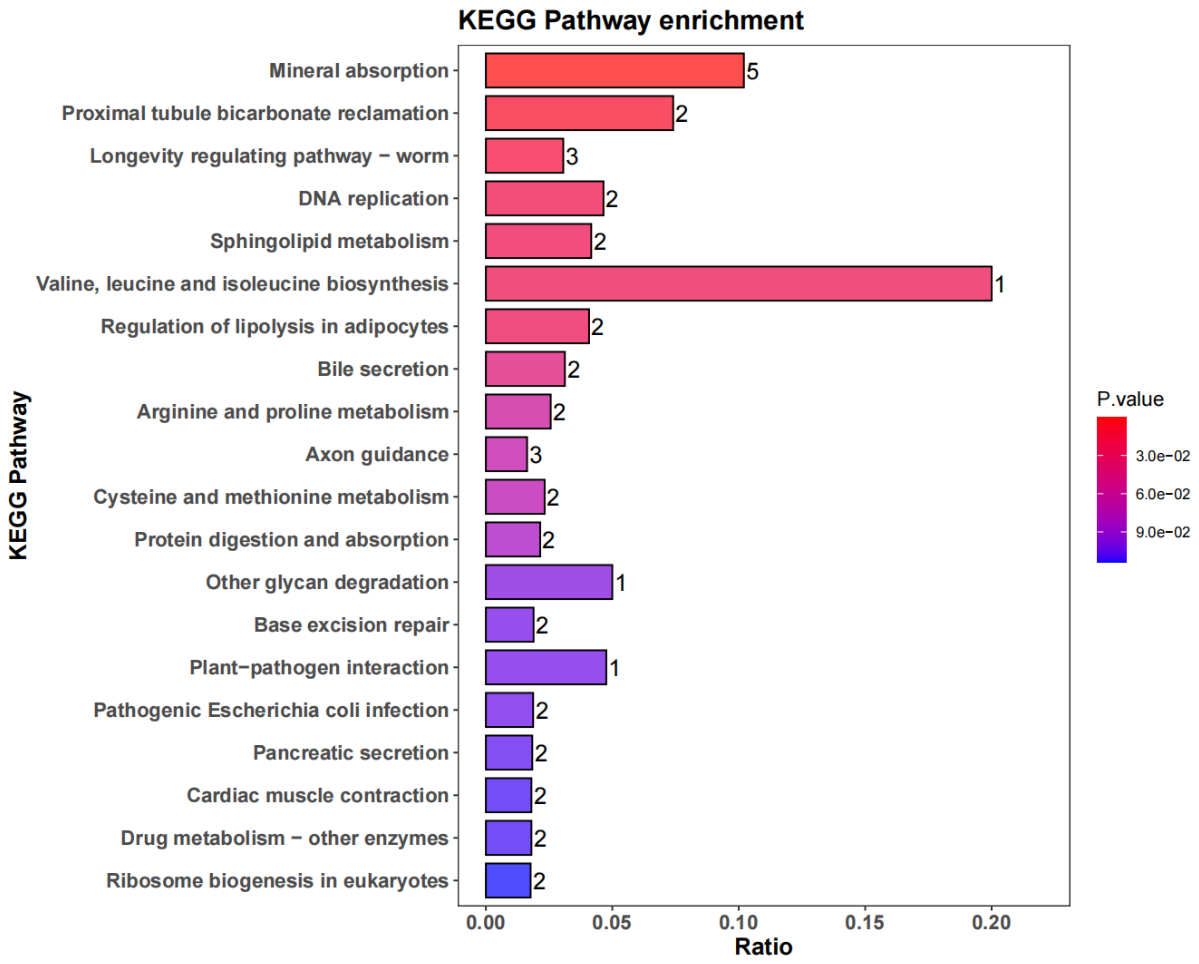

Supplementary Fig. 9. The high-resolution image of Figure 4I.

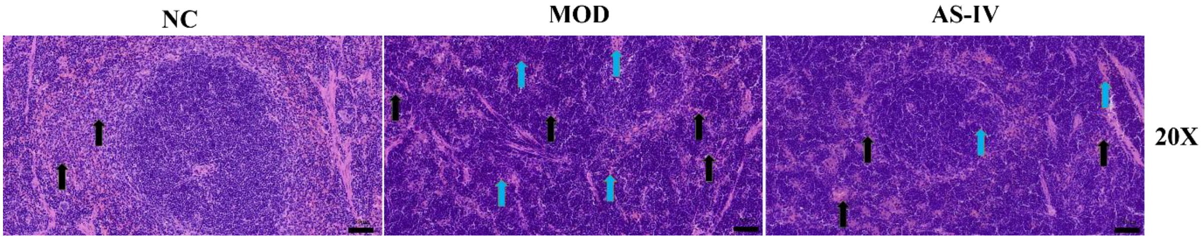

Supplementary Fig. 10. The high-resolution image of Figure 5D.
