# Supplementary figures and images for: Th1/Th2 Immune Imbalance in the Spleen of Mice Induced by Hypobaric Hypoxia Stimulation and Therapeutic Intervention of Astragaloside IV
Source: Int J Mol Sci. 2025 Mar 13;26(6):2584. doi: 10.3390/ijms26062584 (PMC11942621; doi:10.3390/ijms26062584)

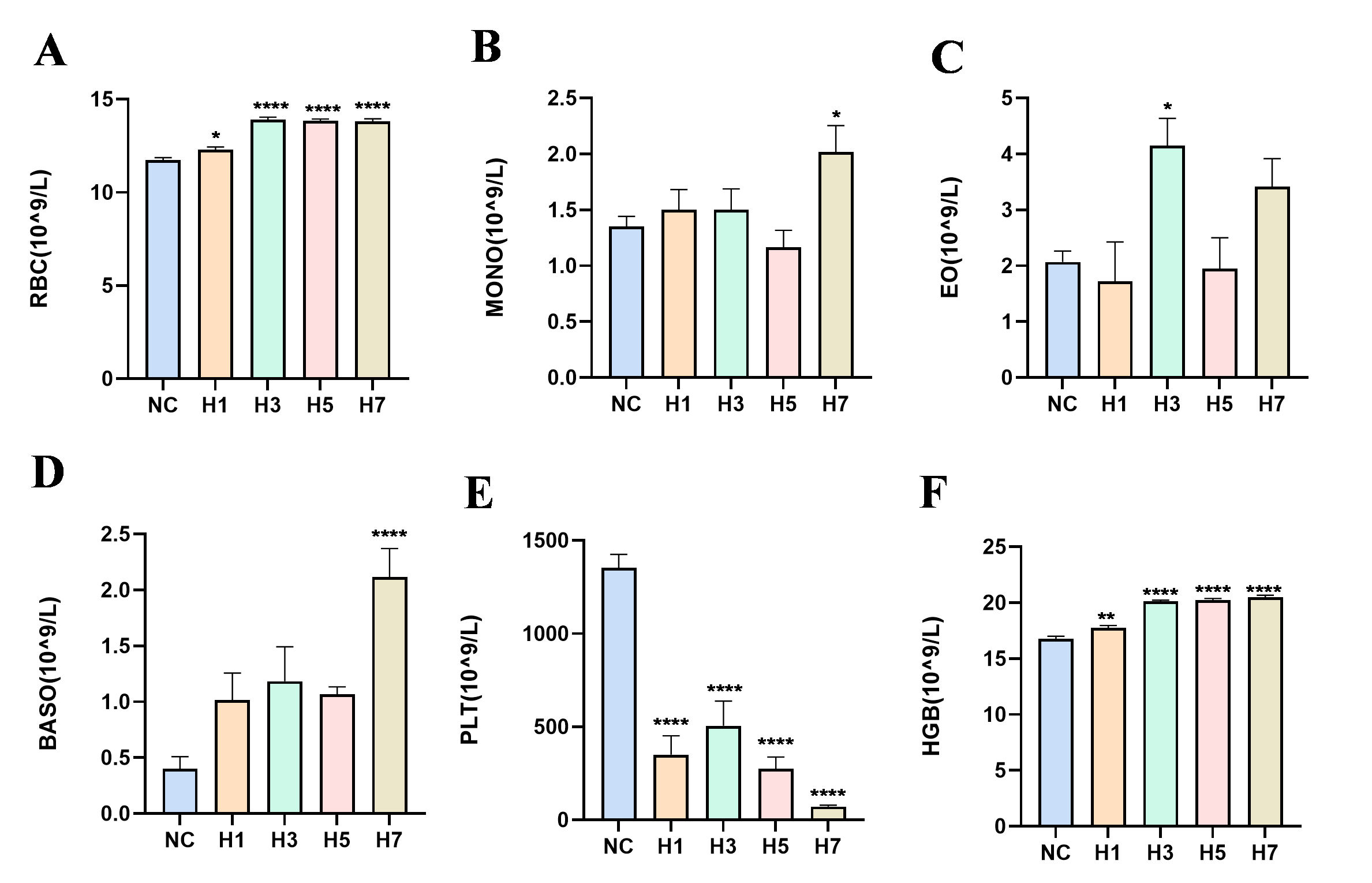

Supplement: Supplementary file 1 [file ijms-26-02584-s001.zip › Supplementary file/Figure S1.tif]

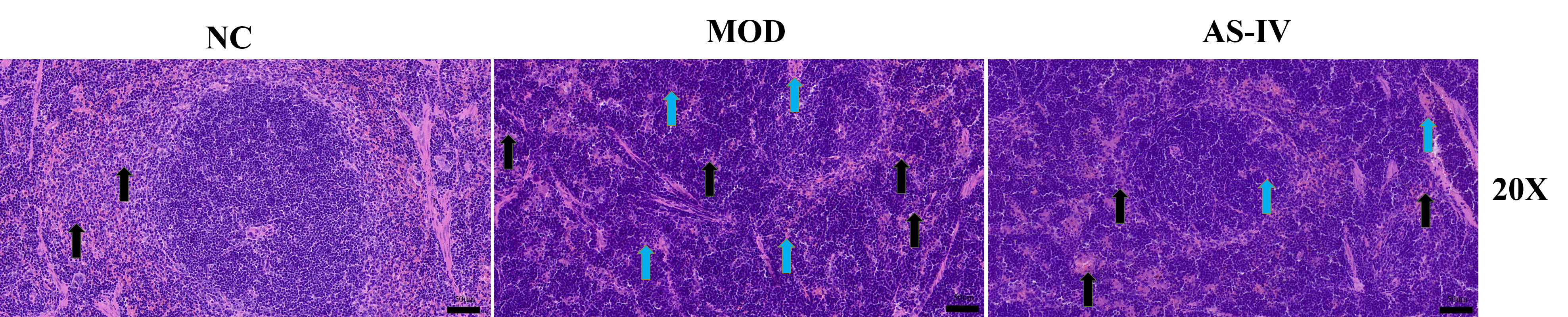

Supplement: Supplementary file 1 [file ijms-26-02584-s001.zip › Supplementary file/Figure S10.tif]

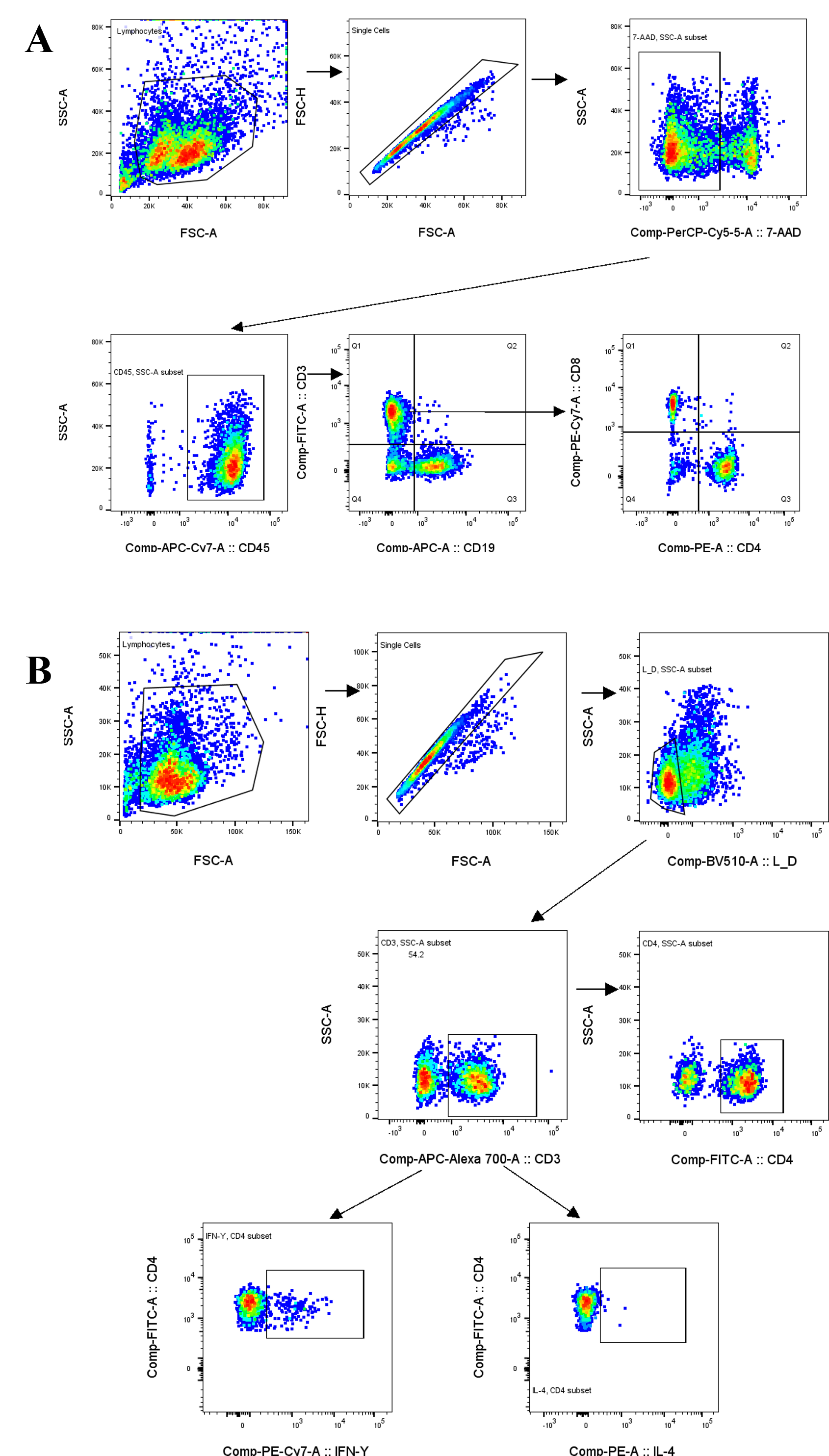

Supplement: Supplementary file 1 [file ijms-26-02584-s001.zip › Supplementary file/Figure S2.tif]

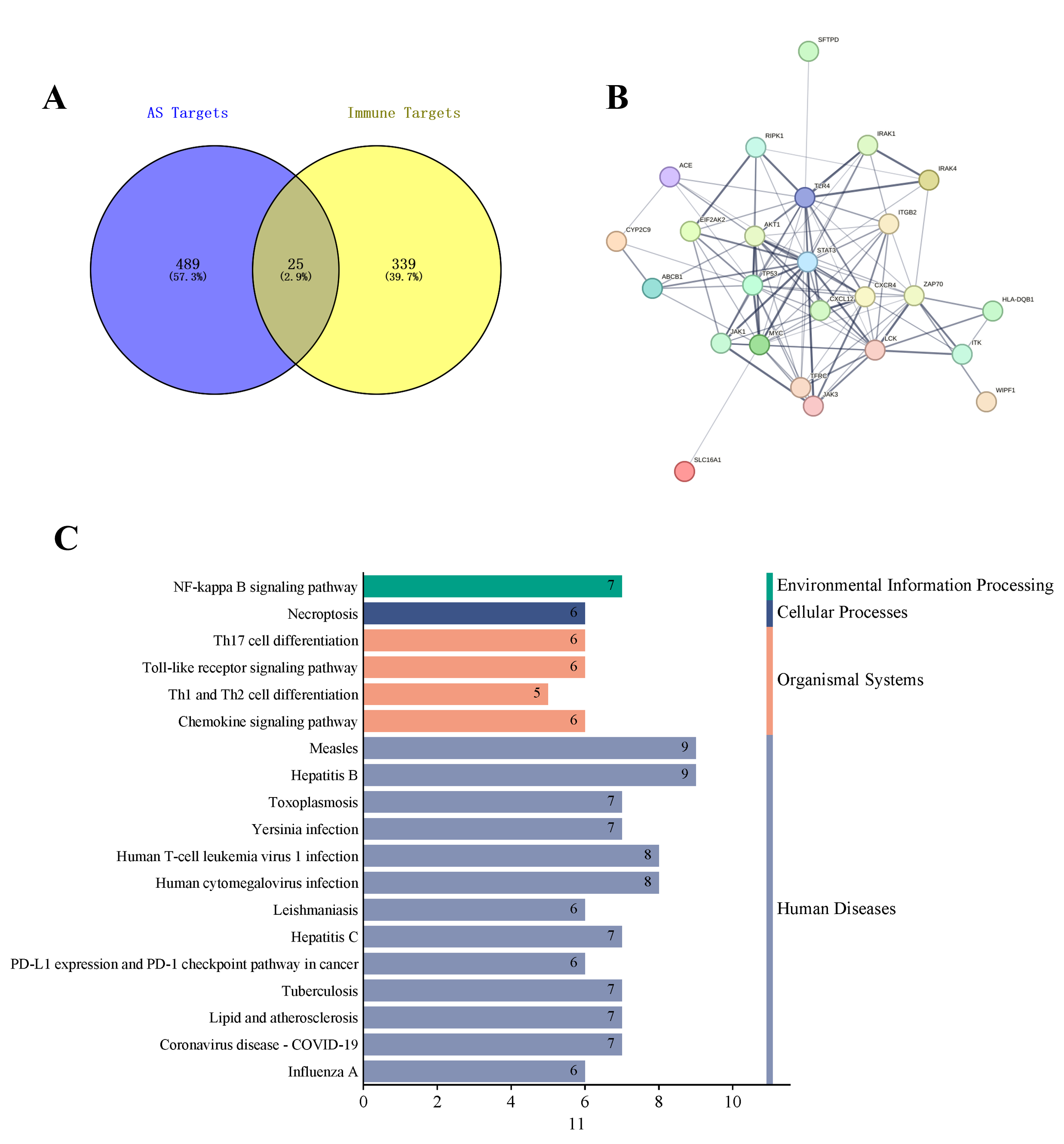

Supplement: Supplementary file 1 [file ijms-26-02584-s001.zip › Supplementary file/Figure S3.tif]

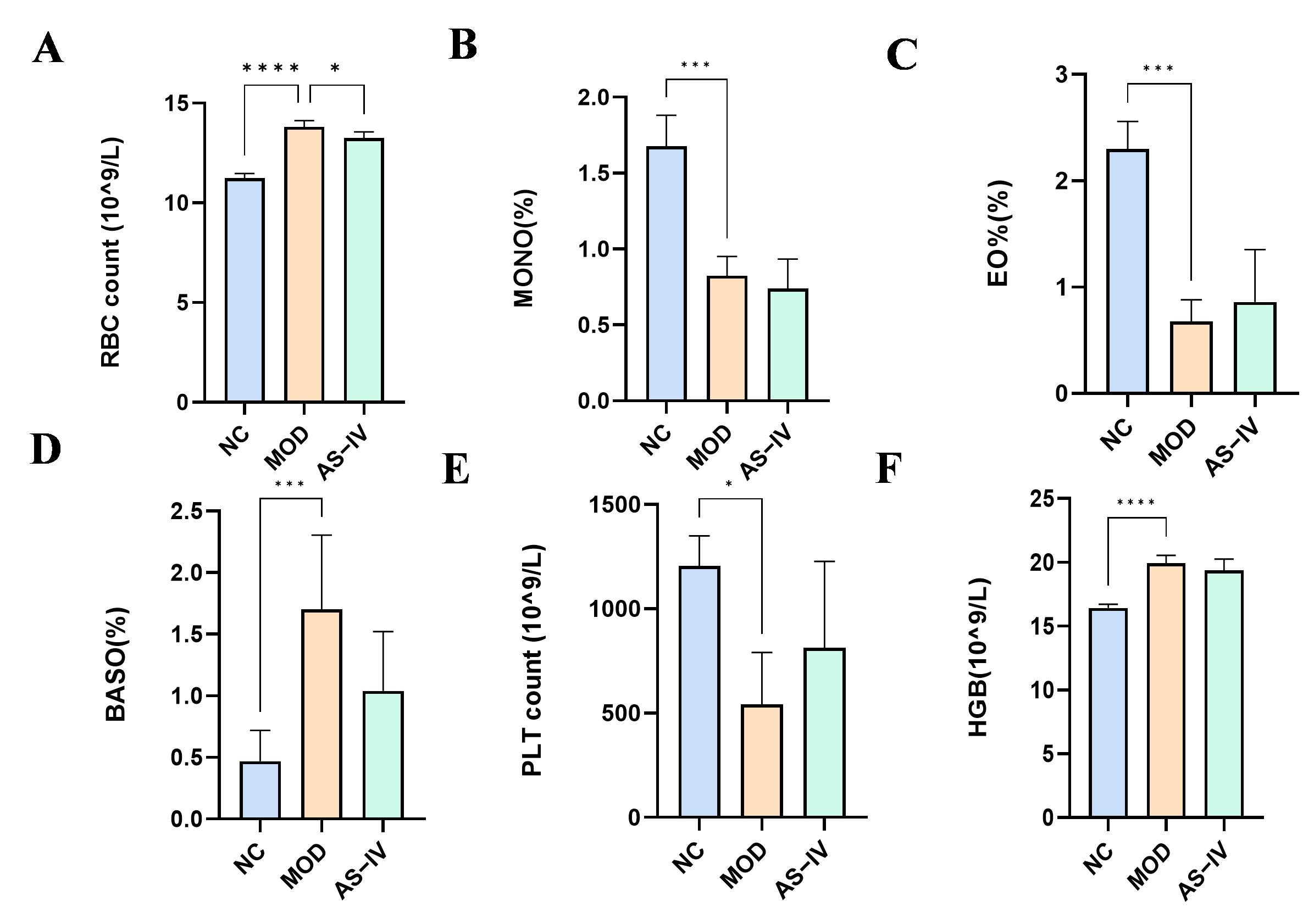

Supplement: Supplementary file 1 [file ijms-26-02584-s001.zip › Supplementary file/Figure S4.tif]

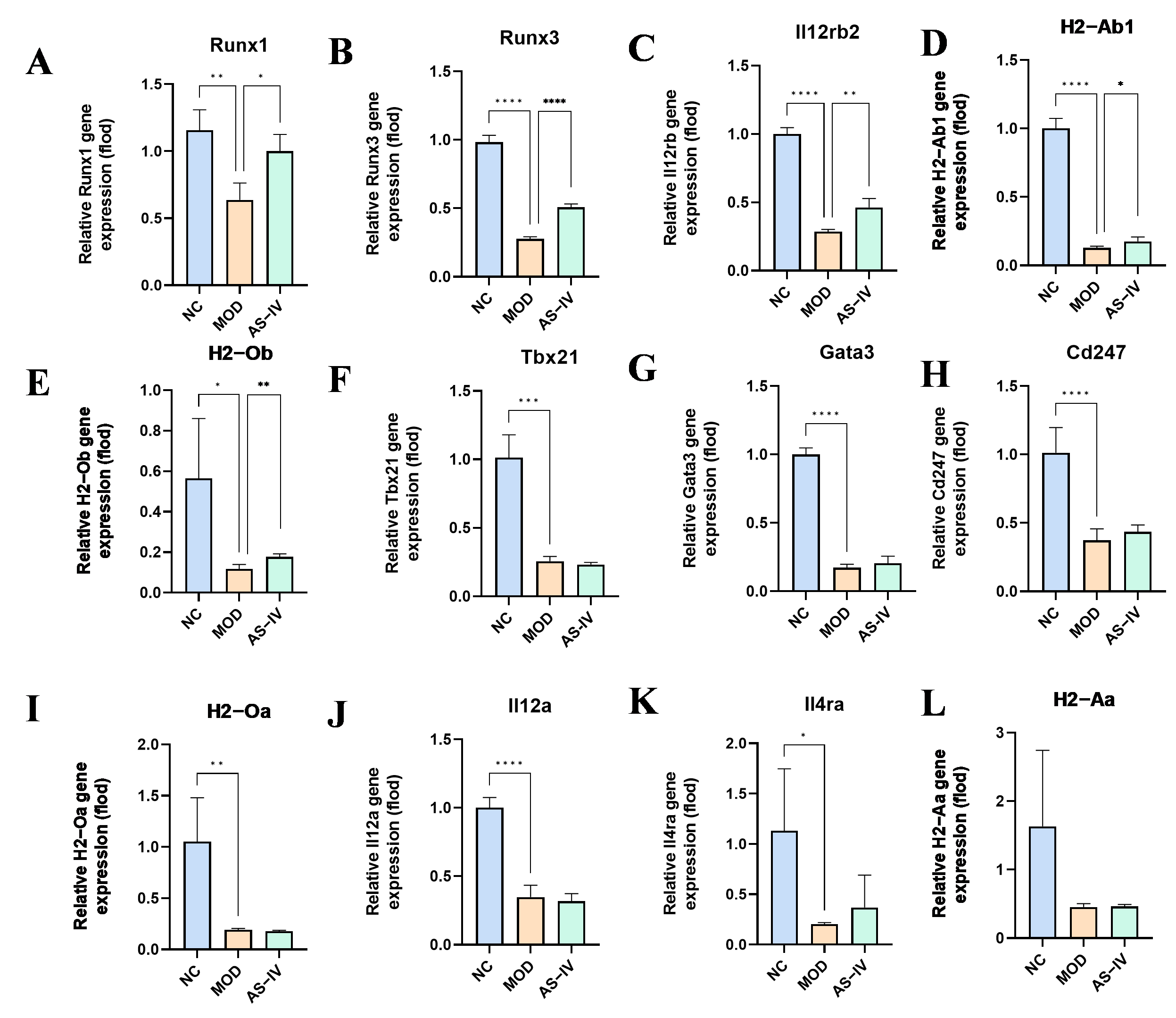

Supplement: Supplementary file 1 [file ijms-26-02584-s001.zip › Supplementary file/Figure S5.tif]

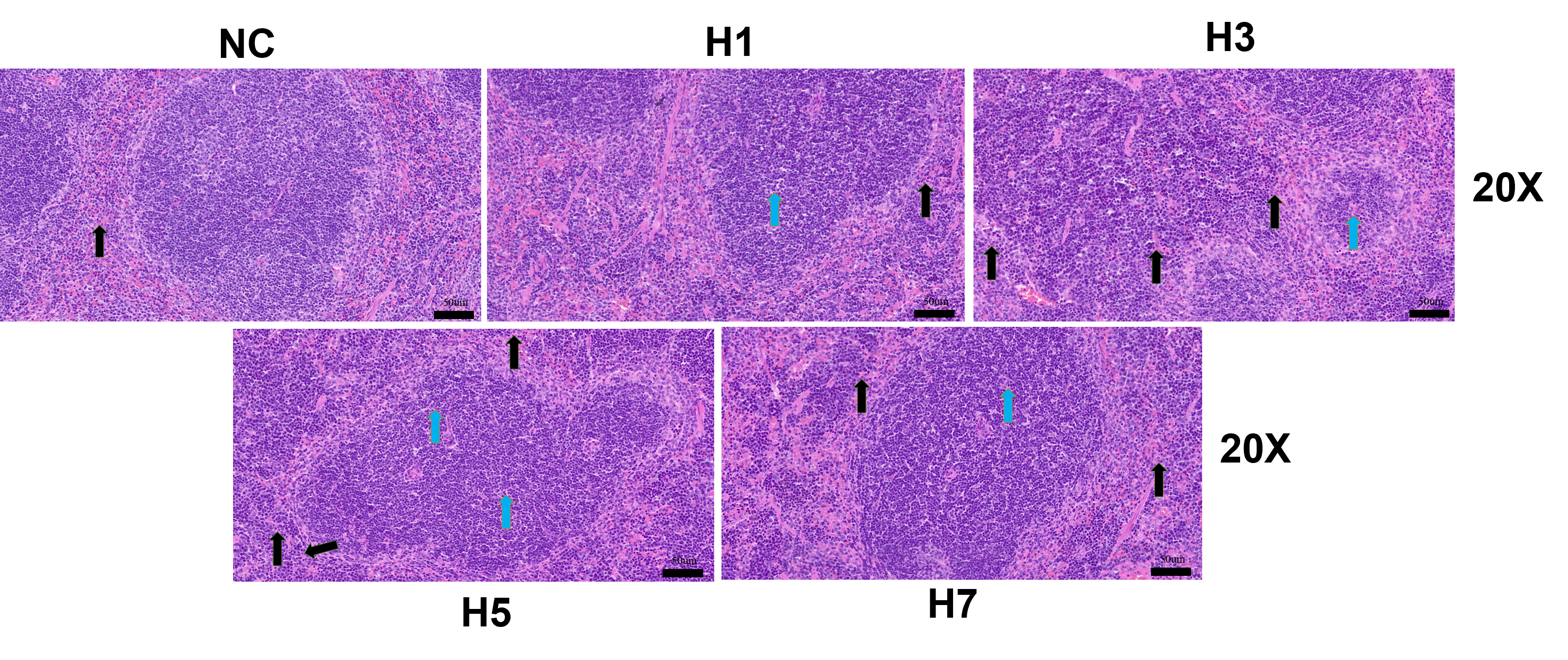

Supplement: Supplementary file 1 [file ijms-26-02584-s001.zip › Supplementary file/Figure S6.tif]

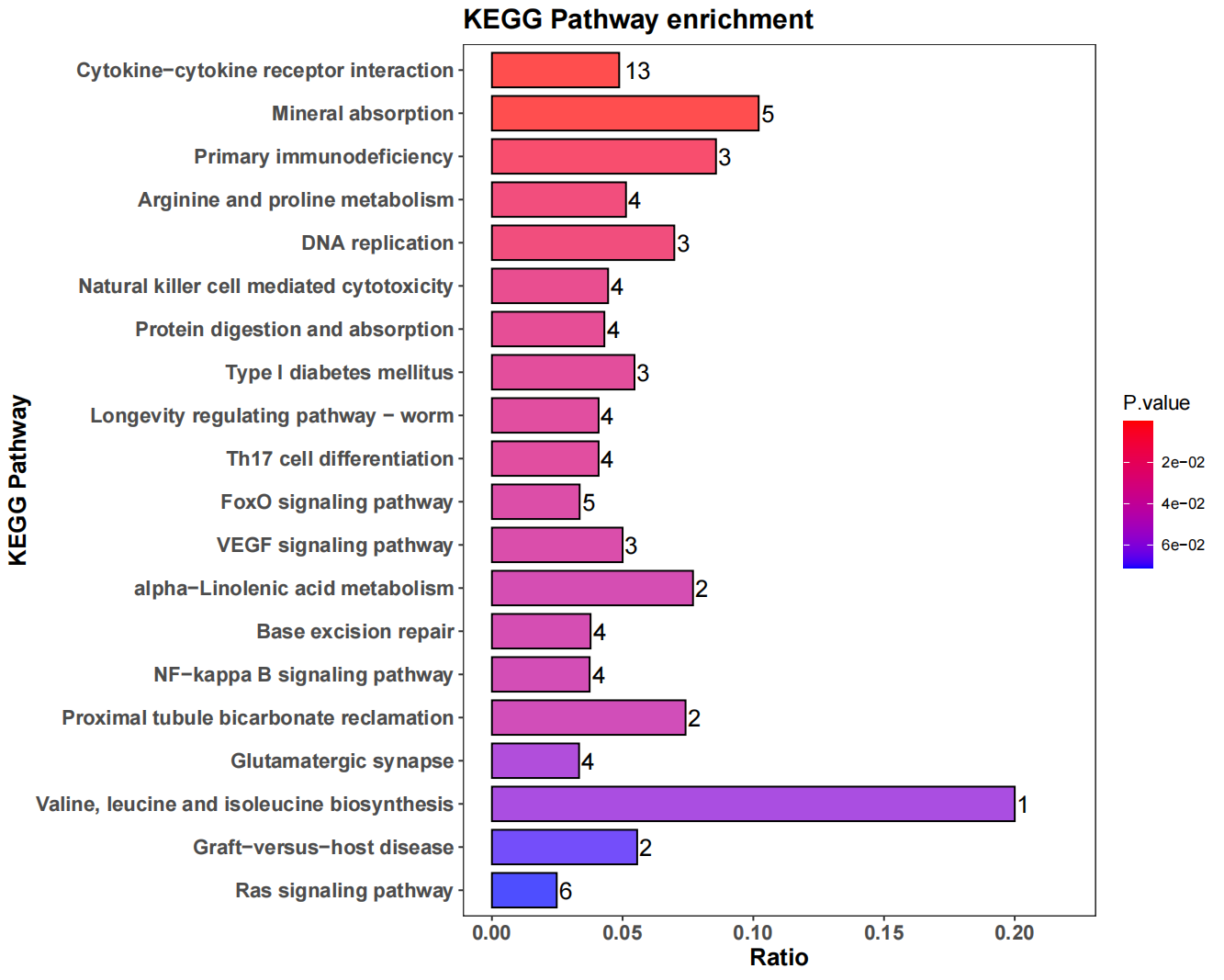

Supplement: Supplementary file 1 [file ijms-26-02584-s001.zip › Supplementary file/Figure S7.tif]

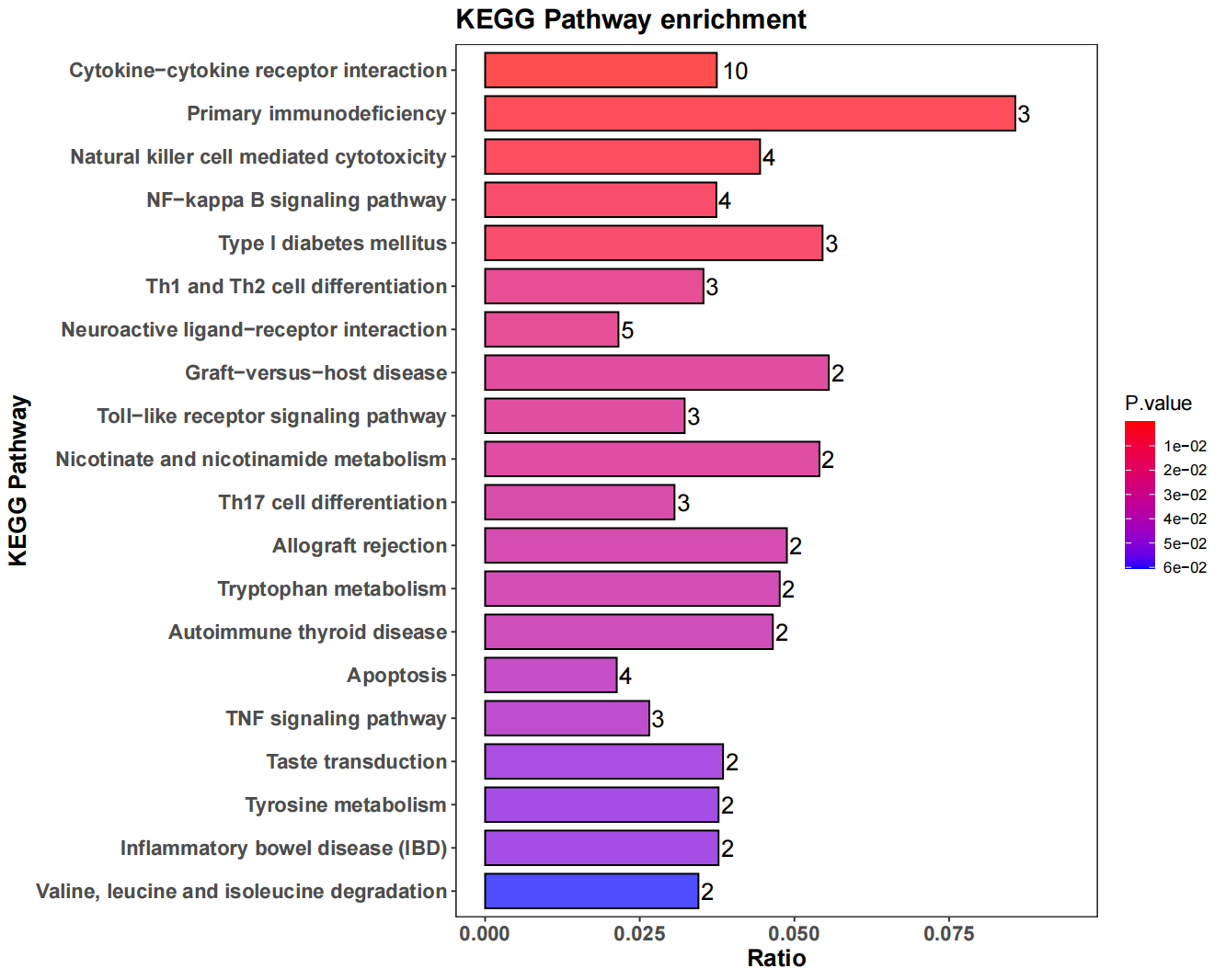

Supplement: Supplementary file 1 [file ijms-26-02584-s001.zip › Supplementary file/Figure S8.tif]

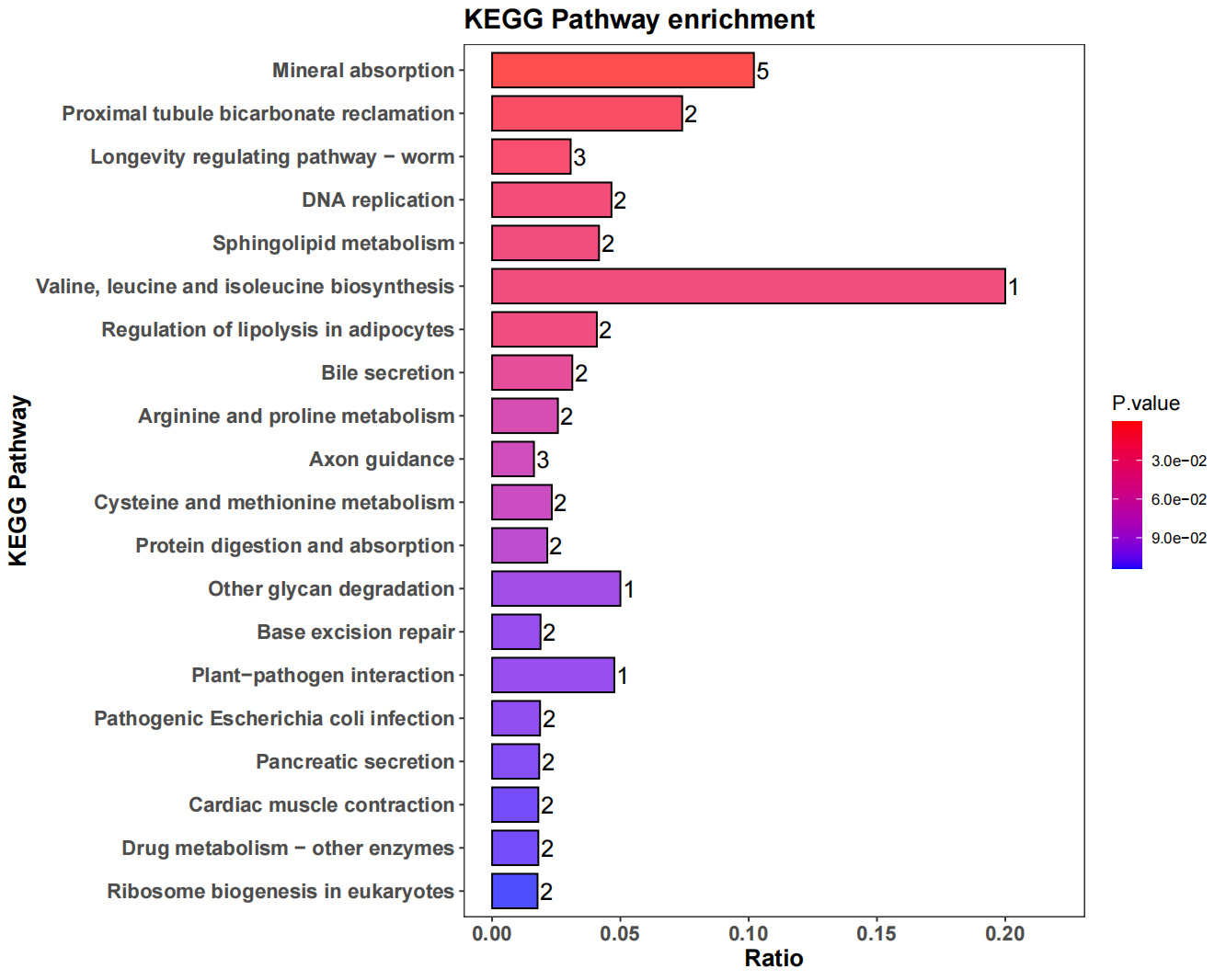

Supplement: Supplementary file 1 [file ijms-26-02584-s001.zip › Supplementary file/Figure S9.tif]
